# Acquiring Resistance Against a Retroviral Infection via CRISPR/Cas9 Targeted Genome Editing in a Commercial Chicken Line

**DOI:** 10.3389/fgeed.2020.00003

**Published:** 2020-05-28

**Authors:** Romina Hellmich, Hicham Sid, Kamila Lengyel, Krzysztof Flisikowski, Antonina Schlickenrieder, Denise Bartsch, Theresa Thoma, Luca D. Bertzbach, Benedikt B. Kaufer, Venugopal Nair, Rudolf Preisinger, Benjamin Schusser

**Affiliations:** ^1^Department of Animal Sciences, Reproductive Biotechnology, School of Life Sciences Weihenstephan, Technical University Munich, Freising, Germany; ^2^Department of Animal Sciences, Chair of Livestock Biotechnology, School of Life Sciences Weihenstephan, Technical University Munich, Freising, Germany; ^3^Institute of Virology, Freie Universität Berlin, Berlin, Germany; ^4^The Pirbright Institute, Woking, United Kingdom; ^5^EW GROUP GmbH, Visbek, Germany

**Keywords:** chicken, genome editing, CRISPR-Cas9, ALV-J, chNHE1

## Abstract

Genome editing technology provides new possibilities for animal breeding and aid in understanding host-pathogen interactions. In poultry, retroviruses display one of the most difficult pathogens to control by conventional strategies such as vaccinations. Avian leukosis virus subgroup J (ALV-J) is an oncogenic, immunosuppressive retrovirus that causes myeloid leukosis and other tumors in chickens. Severe economic losses caused by ALV-J remain an unsolved problem in many parts of the world due to inefficient eradication strategies and lack of effective vaccines. ALV-J attachment and entry are mediated through the specific receptor, chicken Na^+^/H^+^ exchanger type 1 (chNHE1). The non-conserved amino acid tryptophan 38 (W38) in chNHE1 is crucial for virus entry, making it a favorable target for the introduction of disease resistance. In this study, we obtained ALV-J-resistance in a commercial chicken line by precise deletion of chNHE1 W38, utilizing the CRISPR/Cas9-system in combination with homology directed repair. The genetic modification completely protected cells from infection with a subgroup J retrovirus. W38 deletion did neither have a negative effect on the development nor on the general health condition of the gene edited chickens. Overall, the generation of ALV-J-resistant birds by precise gene editing demonstrates the immense potential of this approach as an alternative disease control strategy in poultry.

## Introduction

During the last decades, poultry industry has grown substantially causing difficulties in disease control. Consequently, improving animal welfare while covering an increasing demand for animal protein has become more challenging. Retroviral pathogens continue to be a major problem worldwide. Their relatively high antigenic variability (Kurstak et al., 2013) results in the emergence of new strains and interferes with vaccination-based control strategies (Feng and Zhang, 2016).

The Avian leukosis virus (ALV) is an alpharetrovirus that belongs to the *Retroviridae* family (Lefkowitz et al., 2018). Depending on host range and cross neutralization patterns, the virus is classified into different subgroups (Payne and Nair, 2012). Structural variations of the viral envelope protein underline the evolutionary dynamics responsible for the emergence of new virus strains (Venugopal, 1999). This was illustrated by the identification of ALV-J in the late 80s (Payne, 1998), which increased the number of ALVs that infect chickens to 6 subgroups (ALV A-E and J) (Weiss, 1993). More recently, a putative ALV-K was suspected to be circulating in Chinese indigenous chicken breeds (Wang et al., 2012). The infection with ALV-J can either cause a neoplastic disease or manifests subclinically, which is generally accompanied by reduced weight gain and decreased egg production, leading to high economic losses (Payne and Nair, 2012). In contrast to other ALVs that infect lymphoid cells, commonly causing classical lymphoid leukosis, ALV-J induces a late onset of myeloid leukosis including both myeloblastosis and myelocytomatosis, which is attributed to a distinct cell tropism (Chesters et al., 2002). Since its emergence in 1988, ALV-J became widespread in meat-type chickens worldwide due to highly efficient horizontal transmission and global trade of infected chicken breeding flocks (Zhang et al., 2010; Payne and Nair, 2012). Even though strict eradication programs, similar to those applied for ALV-A and B, were able to partially control ALV-J spread in chickens, subgroup J-related outbreaks are still affecting animal welfare and remain a major threat to poultry industry (Payne and Nair, 2012). In different Asian countries including China, ALV-J is not only endemic (Feng and Zhang, 2016), but also continues to expand the host range and even includes layer-type chickens (Shen et al., 2014); this expanded host range was shown to be associated with increased pathogenicity (Payne and Nair, 2012).

**Figure 1:**
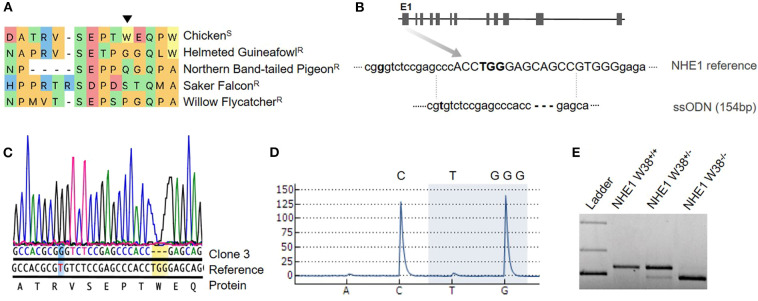
Generation of chNHE1 W38 KO in PGCs and detection of the genetic modification. **(A)** Alignment of the chNHE1 amino-acid sequence with different avian species resistant to AVL-J. The arrow indicates tryptophan (W) at the amino-acid position 38. Susceptible and resistant avian species to ALV-J are marked with (S) and (R), respectively. **(B)** Gene structure of chNHE1 showing the sgRNA target site (capital; www.benchling.com; Off-target 32,9). A single-stranded oligodeoxynucleotide (ssODN), used as a template for homology directed repair (HDR), does not possess the TGG nucleotides and harbors a single nucleotide substitution (T96G, bolt) for the generation of a Bsa1 restriction site (GGTCTC). **(C)** Sanger sequencing chromatogram of the generated NHE1 W38^−/−^ PGC clone 3 compared to chicken NHE1 reference sequence, revealing the successful TGG deletion (yellow background) and T96G substitution (blue background). Protein sequence shows TGG as the W38 encoding nucleotide. **(D)** Detection of lacking TGG nucleotides (highlighted with blue background) in generated NHE1 W38^−/−^ chickens by pyrosequencing. Upper letters represent the part of the NHE1 reference sequence that spans the TGG deletion. Letters below the pyrogram represent the dispension order of the first four subsequently added nucleotides. **(E)** Restriction digest with Bsa1 of the T96G substitution in the edited NHE1 region which was amplified from NHE1 W38^+/+^ birds, heterozygous (NHE1 W38^+/−^) and homozygous (NHE1 W38^−/−^) birds. Separated upper bands were only detected in NHE1 W38^+/−^ based on monoallelic T96G substitution. Full cleavage, leading to a band of reduced size indicates the presence of the biallelic Bsa1 restriction site in NHE1 W38^−/−^ chickens. No Bsa1 cleavage was detected in the NHE1 W38^+/+^ chickens.

ALV infection is initiated by the attachment and subsequent virus entry into the host cell, which requires the interaction of the viral envelope with host cell receptors (Barnard et al., 2006). Cell entry is mediated by a specific cellular receptor and the single determinant of genetic susceptibility to ALV infections and disease. It is long known that birds with specific mutations in the receptor are resistant to ALV infection (Klucking et al., 2002; Elleder et al., 2004). ALV-J is characterized by its unique interaction with a multi-pass transmembrane protein, which was identified as the chicken Na^+^/H^+^ exchanger type 1 (chNHE1) (Chai and Bates, 2006). Among vertebrates, NHE1 is a highly conserved and is ubiquitously expressed. It is involved in essential housekeeping functions including the regulation of intracellular pH (Slepkov and Fliegel, 2002). The ALV-J binding site has been shown to reside within the prominent first extracellular loop (ECL1) of chNHE1, and its structure is defined by the presence of a single amino acid, the non-conserved tryptophan at position 38 (W38, Figure 1) (Kucerova et al., 2013). W38 is exclusively present in ALV-J susceptible species and has been demonstrated to act as key element for virus entry (Kucerova et al., 2013; Lee et al., 2017a).

While natural resistance to ALV-A to E can be seen in inbred chicken lines (Klucking et al., 2002; Elleder et al., 2004, 2005), ALV-J remains an exception (Reinisova et al., 2016). The CRISPR/Cas9 technology has been demonstrated as feasible tool to acquire *in vitro* resistance to ALV subgroups A, B and J using DF-1 cells (Lee et al., 2017a,b; Koslova et al., 2018). Herein, we report the successful introduction of ALV-J resistance in a commercial white leghorn line based on precise deletion of the chNHE1 W38 by gene editing.

## Methods

Detailed methods can be found in Supplementary Material. For the generation of transgenic chickens, primordial germ cells (PGCs) were isolated from a great-grandparent White Leghorn line and expanded for subsequent transfection. As precursors of sperms and oocytes, PGCs play a key role in the establishment of genetic modifications *in vivo* (Sid and Schusser, 2018). In order to introduce the W38 deletion in chNHE1, PGCs were co-transfected with a CRISPR/Cas9 vector expressing a single guide RNA and Cas9-2A-eGFP in combination with a single-stranded oligodeoxynucleotide (ssODN). The ssODN served as DNA repair template to specifically target the W38 coding region of chNHE1 within Exon1 (Figure 1). The designed oligo lacks the W38 coding nucleotides TGG and harbors a T96G nucleotide substitution, which creates a Bsa1 site that can be used later on to detect gene edited birds by PCR followed by restriction enzyme digest (Figure 1).

Forty-eight hour after co-transfection, PGCs were selected with fluorescence activated cell sorting based on transient eGFP expression. Sorted cells were separated by limiting dilution to grow up clonal cell populations. Subsequently, single clones were examined for the defined genetic modification via Sanger and pyrosequencing.

Male chimeric roosters were generated as previously described (Schusser et al., 2013) and raised to sexual maturity. Semen was collected from adult chimeric roosters for gDNA extraction. Sperm analysis was done by pyrosequencing, which has been proven to be a rapid and reliable approach to quantify proportions of mutated and wild-type alleles in various kinds of tissues (Marsh, 2007). The pyrosequencing assay was designed to examine the frequency of modified sperm determined by the presence or absence of the critical TGG nucleotides. Screening for hetero- and homozygosity was performed with pyrosequencing-based genotyping, which was additionally confirmed by Sanger sequencing.

In order to evaluate the ALV-J susceptibility of genetically edited birds, we performed infection experiments using an eGFP transducing modified replication-competent avian sarcoma-leukosis virus with a splice acceptor (RCAS) vector, RCAS(J)eGFP (Kucerova et al., 2013), that was propagated by transfection of DF-1 cells. In three independent infection experiments, we used chicken embryo fibroblasts (CEFs) that were isolated from NHE1 W38^−/−^ and NHE1 W38^+/+^ embryos and infected them with RCAS(J)eGFP virus enriched supernatants. The extent of infection was evaluated by quantification of eGFP expression upon virus replication by flow cytometry.

## Results

Primordial germ cells were transiently co-transfected with the CRISPR/Cas9 vector and the ssODN as repair template. Based on transient eGFP expression of positively transfected cells, 1% highly eGFP positive primordial germ cells were collected by cell sorting (data not shown).

We obtained a total of four clonal PGC lines, all carrying a NHE1 mutation. Both Sanger and pyrosequencing revealed the presence of TGG deletion in three out of four clones (75%), of which two had a biallelic deletion and one was heterozygous. In one of these clones, clone #3, homozygous TGG deletion and T96G substitution occurred simultaneously (Figure 1). This clone was used for the generation of germline chimeras. One clone showed a large deletion of 12 base pairs.

Three chimeras were selected for further breeding based on the relative amount of genetically edited sperm (34, 37, and 46% mutated sperm were identified [data not shown]). All three roosters showed germline transmission (0.4, 2.08, and 0.9% respectively [data not shown]) and gave rise to heterozygous (NHE1 W38^+/−^) offspring.

**Figure 2:**
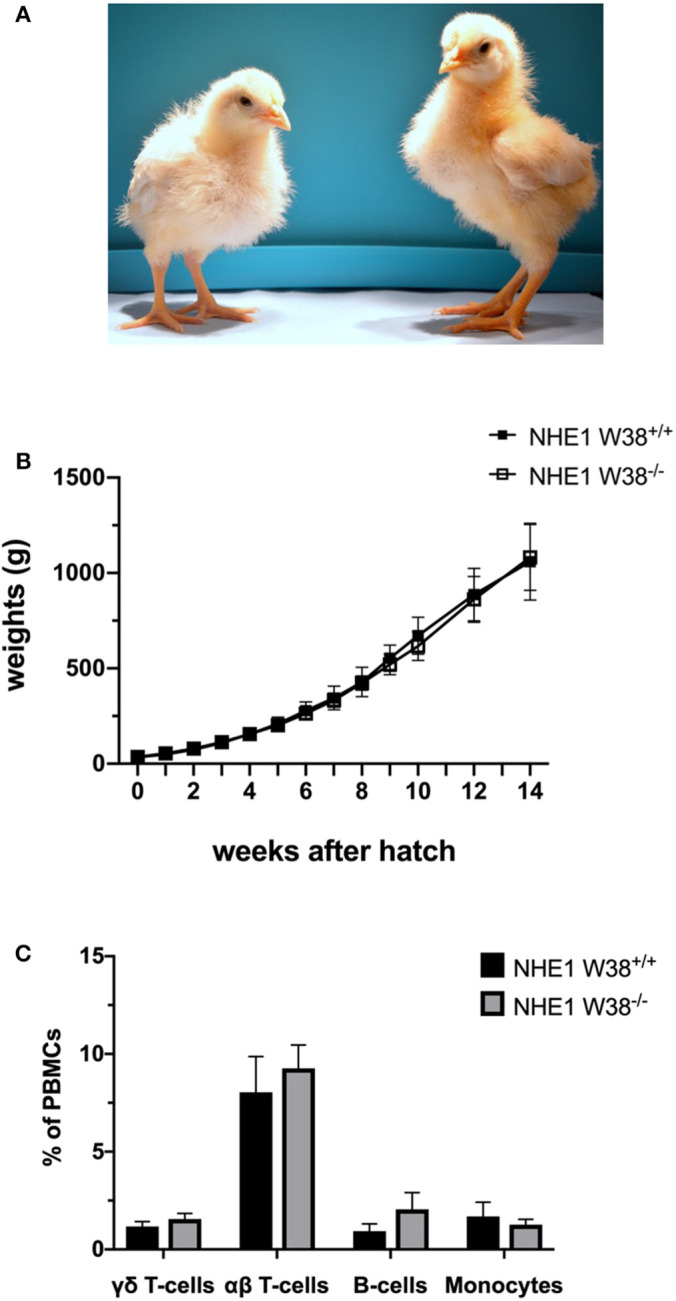
Weight gain and immunophenotype of NHE1 W38^−/−^ birds. **(A)** Representative photo of sibling chicks at 2 weeks of age: NHE1 W38^+/+^ (left) and modified NHE1 W38^−/−^ (right). **(B)** Growth curve of NHE1 W38^+/+^ and NHE1 W38^−/−^ birds. Weight gain was measured weekly from the first day of hatch until 14 weeks of age (*n* ≥ 5). **(C)** Immunophenotype of NHE1 W38^+/+^ and NHE1 W38^−/−^ chickens at 2 weeks of age. Data display relative amounts of T-cells (γδ T-cells: NHE1 W38^+/+^ 1,5%; NHE1 W38^−/−^ 1,8% and αβ T-cells: NHE1 W38^+/+^ 8,0%; NHE1 W38^−/−^ 9,3%), B-cells (NHE1 W38^+/+^ 0,9%; NHE1 W38^−/−^ 2,0%) and monocytes (NHE1 W38^+/+^ 1,7%; NHE1 W38^−/−^ 1,3%), of peripheral blood mononuclear cells (PBMCs) measured by flow cytometry (*n* ≥ 5). Error bars indicate standard deviation (SD). Statistical tests were performed using two-sided Student's *t*-test compared to NHE1 W38^+/+^ birds (*p* > 0.05).

**Figure 3:**
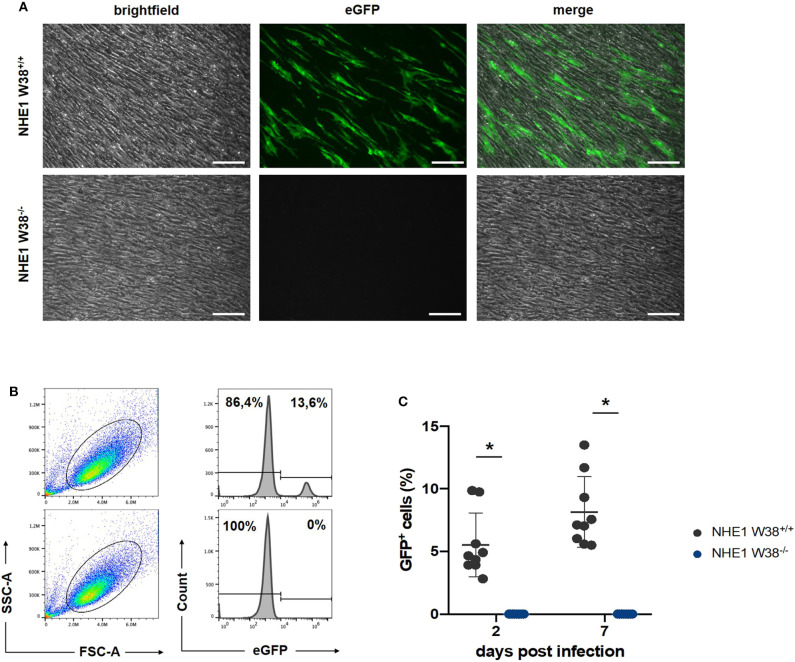
Resistance of NHE1 W38^−/−^ mutants to ALV-J compared to NHE1 W38^+/+^. Infection experiment of NHE1 W38^+/+^ and NHE1 W38^−/−^ chicken embryonic fibroblasts with RCAS(J)eGFP. Infection of the eGFP expressing virus was assessed by microscopy on 14 dpi **(A)** and by flow cytometry on 2 and 7 dpi **(B,C)**. **(A)** Microscopy shows a representative replicate of infected NHE1 W38^+/+^ and NHE1 W38^−/−^ CEFs (Scale bar = 100 μm). **(B)** Flow cytometrical data on 7 dpi. CEFs were gated depending on side- and forward scatter (SSC/FSC) and examined for eGFP fluorescence intensity. Upper panels show infected NHE1 W38^+/+^ and lower panels display NHE1 W38^−/−^ CEFs. **(C)** Comprehensive data of eGFP quantification by flow cytometry on 2 and 7 dpi. Black dots represent NHE1 W38^+/+^ (2 dpi 5.5%; 7 dpi 8.1%) and blue dots NHE1 W38^−/−^ (2 dpi and 7 dpi 0%). Infections were carried out in triplicates in three independent experiments for the indicated cell lines. Standard deviation (SD) is indicated by error bars. Statistical analysis was performed using Mann–Whitney-*U*-Test (*p < 0.05).

In total, seven heterozygous birds were generated to be used for production of homozygous chickens, lacking the critical W38. Detection of biallelic mutations was carried out by pyrosequencing (Figure 1). The modified NHE1 allele was inherited in a Mendelian fashion and the generated NHE1 W38^−/−^ chickens were assessed for possible phenotypic abnormalities (Figure 2) and subsequently examined for their susceptibility to ALV-J (Figure 3).

In order to verify this hypothesis, we examined modified birds for their postnatal development and immunological phenotype. NHE1 W38^−/−^ birds hatched without apparent abnormalities compared to NHE1 W38^+/+^ birds. Upon hatch, NHE1 W38^−/−^ chicks gained weight comparable to NHE1 W38^+/+^ birds (Figure 2). Although, reproduction parameters seem normal, further investigations are needed to determine the role of the mutation in male fertility. Furthermore, the immunophenotype of NHE1 W38^−/−^ birds was analyzed by quantifying the peripheral blood lymphocyte composition using flow cytometry. Here, no significant differences were observed between NHE1 W38^−/−^ and NHE1 W38^+/+^ birds regarding the proportion of monocytes, which are part of the innate immune system, nor in cellular components of the adaptive immune system, represented by B-cells, αβ and γδ T-cells (Figure 2; Supplementary Figure 1).

RCAS(J)eGFP infected NHE1 W38^−/−^ and NHE1 W38^+/+^ derived CEFs with RCAS(J)eGFP were cultured and screened for eGFP expression using fluorescence microscopy at 14 days post infection (dpi) (Figure 3). In addition, we quantified the infection level on 2 and 7 dpi by flow cytometry detecting eGFP fluorescence. No infection was observed in NHE1 W38^−/−^ CEFs, while it was able to infect NHE1 W38^+/+^ CEFs at 2 and 7 dpi with 5.6 and 8.1%, respectively (Figure 3).

## Discussion

Our findings demonstrate that cells from NHE1 W38^−/−^ transgenic chicken are completely resistant to ALV-J infection. This is consistent with previous studies, which emphasized resistance based on W38 related deletions in chicken cell lines (Kucerova et al., 2013; Lee et al., 2017a; Koslova et al., 2018). Based on the natural occurring mutation (NHE1ΔW38) in ALV-J resistant birds (Kucerova et al., 2013), we generated chickens carrying the same genetic modification in a commercial great-grandparent White Leghorn line, which led to a complete resistance against ALV-J, as indicated by the abrogation of the cell-pathogen binding of RCAS(J)eGFP. Here, a retroviral vector which contains the ALV-J *env* gene served as a representative tool to determine susceptibility to infection with ALV-J, as previously described (Kucerova et al., 2013).

Since maintaining NHE1 integrity is vital for cell physiology (Slepkov and Fliegel, 2002), it was a prerequisite to preserve the NHE1 function in the generated animals. Available data from gene edited mice indicate that atypical NHE1 activity is associated with physiological disorders (Bell et al., 1999). NHE1 mutant mice exhibited a decreased postnatal growth, increased mortality and disorders of the central nervous system. This phenotype was induced by targeted disruption of several amino acids within highly conserved domains, indicating their critical importance for essential NHE1 functions. In contrast, we modified the first extracellular loop 1—a region, which has been shown to be functionally not dependent on high structural homogeneity (Shrode et al., 1998). This was done by deleting the single amino acid W38 of NHE1. Additionally, it was shown that W38-related deletions and substitutions naturally occur among avian species (Kucerova et al., 2013). Hence, the present modification in chickens is unlikely to interfere with physiological activity of NHE1 *in vivo*. Our phenotyping data confirmed this assumption of W38 being dispensable for NHE1 function *in vivo*.

The relevance of ALV-J as a constant threat for animal health worldwide is further supported by recent epidemiological research. Field studies report a broader spectrum of avian species to carry ALV-J susceptible alleles and thereby function as potential site of virus multiplication beside chicken, turkey and the red junglefowl (Plachy et al., 2017). Additionally, wild birds might be considered as potential reservoir for ALV-J, which was supported by the identification of ALV-J isolates among *Anseriformes* and *Passeriformes* (Jiang et al., 2014; Zeng et al., 2014).

During the process of finalizing this manuscript, Koslová et al. conducted a similar study (Koslova et al., 2020). The authors were able to induce a NHE1 W38 mutation in a CB line, which is an inbred chicken line characterized by a specific MHC-haplotype, known to affect susceptibility to viral diseases such as Marek's Disease Virus or Rous sarcoma virus (Miller and Taylor, 2016). The CB inbred line shows deficits in reproduction and in its general health constitution (Aumann, 2017). As previously reported, hens were artificially inseminated to increase the probability of fertilization (Koslova et al., 2020). This could be considered as a non-conventional way in the breeding practices of poultry production. Our study is the first work reporting the possibility of inducting the W38 mutation in livestock using relevant layer type chickens. Obtaining NHE1 W38^−/−^ healthy birds leave no doubt that this mutation does not cause a pathological phenotype in chicken, which is impossible to fully investigate in the CB line due to their divergent physical constitution.

Despite of immense efforts that were spent on the development of effective vaccines against ALV-J, the virus continues to cause high economic losses and may evolve to increased pathogenicity (Feng and Zhang, 2016). Our genetically engineered NHE1 ΔW38 chicken line provides a valid alternative to reach ALV-J resistance compared to present breeding strategies that often lack effectiveness due to missing resistance alleles among affected populations (Reinisova et al., 2016; Whitworth et al., 2016). The direct introduction of disease resistance might be a favorable option along with intensive eradication programs or preventive treatment based on vaccines.

The availability of genome-editing tools, notably CRISPR/Cas9, widens the scope of animal breeding and its applications in the context of disease control (Sid and Schusser, 2018). By generating an ALV-J resistant chicken line, we provide an efficient and valuable model for further gene-engineering in livestock, which may open new perspectives in disease control to improve animal welfare.

## Data Availability Statement

The datasets generated for this study are available on request to the corresponding author.

## Ethics Statement

Animal experiments were approved by the Government of Upper Bavaria, License number 2018-09-23 ROB-55.2-2532.Vet_02-18-9.

## Author Contributions

BS and RP conceived and planned the project. BS, RH, HS, KL, VN, KF, BK, and LB designed the experiments. RH, HS, AS, and DB performed all experiments. TT, RH, HS, and KL analyzed the data and RH, HS, and BS wrote the manuscript.

## Conflict of Interest

RP was employed by the company EW group GmbH. The remaining authors declare that the research was conducted in the absence of any commercial or financial relationships that could be construed as a potential conflict of interest.
