# Supplementary material for: Acquiring Resistance Against a Retroviral Infection via CRISPR/Cas9 Targeted Genome Editing in a Commercial Chicken Line
Source: Front Genome Ed. 2020 May 28;2:3. doi: 10.3389/fgeed.2020.00003 (PMC8525359; doi:10.3389/fgeed.2020.00003)
Supplement: Supplementary file 1 [file Table_1.DOCX]

Supplementary Material

Animals

Eggs from a commercial great-grandparent White Leghorn line (LSL) were obtained from Lohmann-Tierzucht GmbH (Cuxhaven, Germany). Chickens were housed in S1 animal facility designed for genetically modified chickens at the TU Munich (School of Life Sciences, Weihenstephan TU Munich). Water and commercial feed were provided ad libitum. Animal experiments were conducted according to current law and were approved by the government of Upper Bavaria (experiment license ROB-55.2-2532.Vet_02-18-9).

Germline chimeras and genetically modified birds were generated as previously described [34]. Briefly, chimeric roosters were screened for the presence of modified sperm based on sperm-DNA analysis via pyrosequencing. Homozygous birds were generated by crossing the heterozygous birds (NHE1 W38^+/-^).

Derivation and culture of cells

LSL PGCs were derived from blood of embryonic vasculature at stages 13-15 according to Hamburger and Hamilton as described before [35]. PGCs were derived from male embryos and cultured at 37°C in 5% CO_2_ environment using modified KO-DMEM as described previously [36].

Chicken embryonic fibroblasts (CEFs) were isolated according to a previously established protocol [37] from chNHE1 W38^+/+^ and NHE1 W38^-/-^ embryos on the 10^th^ embryonic day (ED). Prior to isolation of CEFs, genotyping of embryos was done by collecting blood at ED10 preparing a window of 0,5cm^2^ in the eggshell that allowed to access the embryonic vasculature. CEFs were cultured using Iscove's liquid medium containing stable glutamine (Biochrom, Germany), and was supplemented with 5% fetal bovine serum (FBS) Superior (Biochrom, Germany), 2% chicken serum (ThermoFisher Scientific, USA) and 1% Penicillin-Streptomycin-Solution (Penicillin 10,000 U/ml and Streptomycin 10 mg/ml) (Biochrom. Germany). Subsequently, CEFs were incubated at 40°C in 5% CO_2_ atmosphere on 100x17mm petri dishes (ThermoFisher Scientific) until infection.

The DF-1 fibroblast cell line was obtained from the Federal Research Institute for Animal Health (FLI Riems, Germany). Cells were maintained in DMEM supplemented with 10% FBS and 1% Glutamax and were incubated at 40°C, 5% CO_2_.

Gene-editing in Primordial Germ Cells (PGCs)

A single guide RNA (sgRNA) targeting the first exon of the chicken NHE1 gene was designed (Suppl. table 1) using the following website: <https://benchling.com/crispr> and cloned into the vector backbone, pSpCas9(BB)-2A-eGFP (PX458) (Addgene, USA), that contains eGFP as a selectable marker. A single-stranded oligodeoxynucleotide (ssODN) was synthesized (Suppl. table 1) by Integrated DNA Technologies (IDT, USA) to serve as a repair template [18].

For transient transfection of PGCs, a total of 5x10^6^ cells were washed with phosphate-buffered saline (PBS) and resuspended in 100µl premixed transfection solution, containing 10µg targeting vector (PX458-sgRNA), 10µg ssODN in Nucleofector^TM^ Solution V (Lonza, Germany). Electroporation was performed using an ECM 830 Square Wave Electroporation System (BTX, USA), applying eight square wave pulses (350V, 100µsec). Subsequently, the cell suspension was mixed with 500µl PGC Medium and incubated for 5 min at 37°C to allow cell recovery. Finally, cells were resuspended in the appropriate amount of culture medium and cultured under regular conditions for the following 48h. Next, the transfected cell pool was prepared for fluorescence activated cell sorting (FACS) using BD FACSAriaTM Fusion Cell Sorter (BD Biosciences, USA). Briefly, PGCs were washed with PBS and resuspended to 2,5x10^6^ cells/ml in CO_2_ independent Medium (ThermoFisher Scientific, USA), including 10% FBS and 1% Glutamax (ThermoFisher Scientific, USA) and sorted based on eGFP fluorescence. Selected eGFP-positive PGCs were plated on a 48-well plate in limiting dilution in order to expand single cell clones.

RCAS(J)eGFP propagation and infection of CEFs

A modified retroviral RCAS(J)eGFP vector was used for infection experiments [17]. The vector was generated by replacing the RCAS envelope with the original sequence of the ALV-J (HPRS-103 strain) envelope gene. In order to propagate RCAS(J)eGFP-virus for infection experiments, DF-1 cells were transfected with 500ng RCAS(J)eGFP-DNA using ViaFect^TM^ Transfection Reagent (Promega, USA) at a 6:1 ratio of ViaFect™ Transfection Reagent:DNA, according to the manufacturer’s instructions. 32 days post transfection, RCAS(J)eGFP-virus enriched supernatant was collected, centrifuged for 10 min at 1000 x g at 4 °C and subsequently stored at -80°C for further experiments.

24h prior to RCAS(J)eGFP infection, CEFs were seeded into 6-well plates (1x10^5^ per well). CEFs were infected using 100µl of RCAS(J)eGFP enriched supernatant, while control wells were kept uninfected.

Genotyping and Pyrosequencing

Sanger Sequencing was performed in order to confirm the NHE1 W38 mutation and T96G substitution. Genomic DNA was extracted from cell pellets or blood using ReliaPrep™ Blood gDNA Miniprep System (Promega, USA). PCR amplification of the targeted NHE1 region was done with 5x FIREPol® Master Mix (Solis BioDyne, Estonia) and gene specific primers (Suppl. Table 1), using an annealing temperature of 59,4°C. The resulting amplicon was gel-purified using E.Z.N.A.® Ultra-Sep® Gel Extraction kit (OmegaBiotek, USA) and subsequently cloned into pGEM®-T Easy Vector System I (Promega, USA) according to the manufacturer’s instructions. Plasmid DNA was extracted by PureYield™ Plasmid Miniprep System (Promega, USA) and sent for Sanger Sequencing (Eurofins, Germany).

A restriction enzyme digest was conducted in order to track the presence and functionality of the introduced Bsa1 restriction site. gDNA from blood of the generated transgenic birds was isolated to amplify the NHE1 region harbouring the Bsa1 site using primer #1 and 2 (Suppl. table 1) and 5 x FIREPol® Master Mix (Solis BioDyne, Estonia). 600ng of the resulting amplicon were digested with 10U Bsa1 (New England Biolabs, USA) for 1h at 37°C and subsequently analysed by gel electrophoresis.

Pyrosequencing was carried out for allelic quantification of the TGG deletion in sperm of germline chimeras and the detection of modified offspring. For that purpose, a PCR was performed to generate a sequencing template, using 5x FIREPol® Master Mix and gene specific primers (Suppl. table 1). The pyrosequencing reaction was performed based on the resulting amplicon using PyroMark Q48 Advanced Reagents (4 x 48) and PyroMark Q48 Autoprep (QIAGEN, Germany). A sequencing primer was designed, to bind upstream of the induced TGG deletion, that allows sequence analysis of C[TGG]GAGCAGCCG (primer #4, Suppl. table 1). Both assays, AQ (allelic quantification) and SNP (genotyping), were designed via PyroMark Assay Design Software (QIAGEN, Germany). Data were analysed by PyroMark Q48 Autoprep 2.4.2 software (QIAGEN, Germany).

FACS analysis

Susceptibility of modified CEFs and WT CEFs to RCAS(J)GFP was determined by flow cytometry. Cells were detached using 1xTrypsin/EDTA Solution, washed with PBS and resuspended in Fluo-Buffer (PBS containing 1% Bovine Serum Albumin (Sigma-Aldrich, USA) + 0,01% NaN_3_-solution (Applichem, Germany) to a final concentration of 2x10^6^ cells/ml.

The immunophenotype of genetically modified birds was analysed based on the number of peripheral blood mononuclear cells (PBMCs). Cells were isolated from blood of 14 days-old chickens by standard density gradient centrifugation (Biocoll separating solution, Biochrom, Germany). A total of 1x10^6^ PBMCs per well were plated on a 96-well round bottom plate and stained for 20min on ice, applying following unlabelled primary antibodies (Southern Biotech, USA): Mouse anti-chicken TCRγδ (TCR-1), mouse anti-chicken TCRαβ/Vβ1 (TCR-2) + mouse anti-chicken TCRαβ/Vβ2 (TCR-3), mouse anti-chicken Bu1 (AV20), mouse anti-chicken KUL01 (KUL01). Unbound antibodies were removed by washing with PBS. Next, cells were incubated for 20 min on ice with goat anti-mouse IgG(H+L)-APC (Southern Biotech, USA). In a final step, cells were washed with PBS and resuspended in 400µl Fluo-Buffer.

Fluorescence was measured using BD Accuri™ C6 flow cytometer (BD Biosciences, USA). Gating strategy is provided in supplementary figure 1. Data was analysed with FlowJo 10.4.1 software (FlowJo, LLC 2006-2017, USA).

Statistical analysis

Statistical analysis was carried out using SPSS24 statistics (version 24.0.0.0) software (IBM, USA). Normally distributed data (Shapiro-Wilk test *p* > 0,05) were analysed by student´s T-Test. The Mann-Whitney-U Test was applied for not normally distributed data.

Graphs were constructed using GraphPad Prism (version 8.0.1 145) (GraphPad Software, USA).

| **Table 1. List of designed ssODN and oligos** | | | |
| --- | --- | --- | --- |
| **Construct name** | **Type** | **Purpose** | **Sequence (5´ - 3´)** |
| chNHE1 sgRNA | single guide RNA | CRISPR/Cas9 targeting | ACCTGGGAGCAGCCGTGGGG  (PAM: CCC) |
| chNHE1 ssODN | repair construct | HDR | GCCCGCTGCTGCCCGGCCAGCGCTTGCAGGCCGACGCCACGCGGGTCTCCGAGCCCACCGAGCAGCCGTGGGGAGAGCCCGGGGGTATCACCGCCGCCCCGCTGGCCACGGCCCAGGAGGTGCACCCGCTGAACAAACAGCACCACAACCACTC |
| P1  (chNHE1  5´flanking region) | primer (forward) | Sanger Sequencing, Bsa1 treatment | GCACCTCACGCCTGTGCAAC |
| P2  (chNHE1-Exon1) | primer (reverse) |  | GGGATGCGGACGTGCGAGTA |
|  |  |  |  |
| P3  (chNHE1-Exon1) | primer (forward) | Pyrosequencing | CCTTCCCTGGGCTCTGCT |
| P4  (chNHE1-Exon1) | primer (reverse, biotinylated) |  | [BIOT]GATACCCCCGGGCTCTCC |
| P5  (chNHE1-Exon1) | primer (forward) |  | GTCTCCGAGCCCAC |
|  |  |  |  |
