# Supplementary figures and images for: Acquiring Resistance Against a Retroviral Infection via CRISPR/Cas9 Targeted Genome Editing in a Commercial Chicken Line
Source: Front Genome Ed. 2020 May 28;2:3. doi: 10.3389/fgeed.2020.00003 (PMC8525359; doi:10.3389/fgeed.2020.00003)

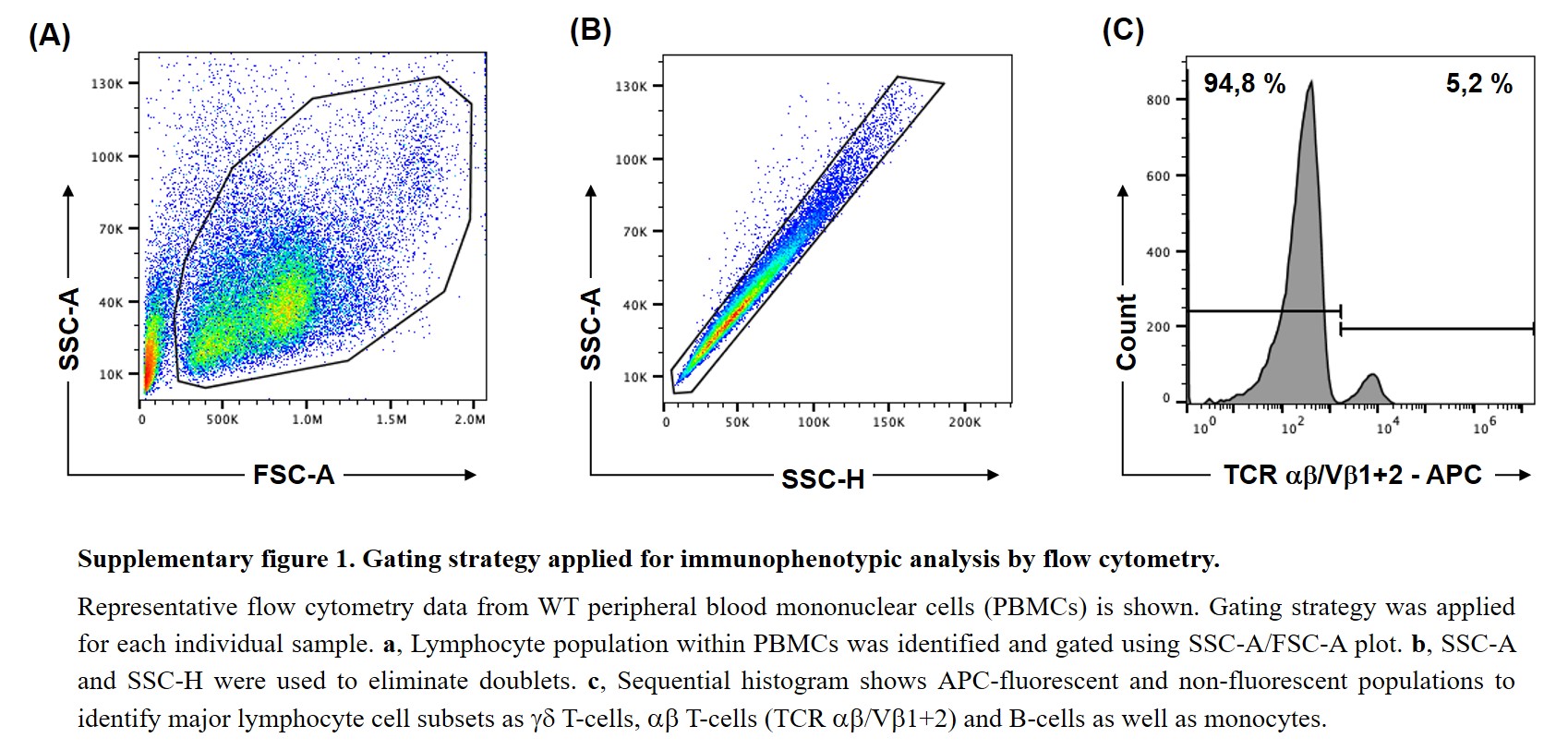

Supplement: Supplementary file 2 [file Image_1.JPEG]
